# Pharmacist provision of primary health care: a modified Delphi validation of pharmacists' competencies

**DOI:** 10.1186/1471-2296-13-27

**Published:** 2012-03-28

**Authors:** Natalie Kennie-Kaulbach, Barbara Farrell, Natalie Ward, Sharon Johnston, Ashley Gubbels, Tewodros Eguale, Lisa Dolovich, Derek Jorgenson, Nancy Waite, Nancy Winslade

**Affiliations:** 1 Summerville Family Health Team, Mississauga, Ontario, Canada; 2 C.T. Lamont Primary Health Care Research Centre, Élisabeth Bruyère Research Institute, Ottawa, Ontario, Canada; 3 Department of Sociology and Anthropology, University of Ottawa, Ottawa, Ontario, Canada; 4 Clinical and Health Informatics Research Group, Department of Epidemiology, Biostatistics and Occupational Health, McGill University, Montreal, Quebec, Canada; 5 Department of Family Medicine, McMaster University, Centre for Evaluation of Medicines, Hamilton, Ontario, Canada; 6 College of Pharmacy and Nutrition, University of Saskatchewan, Saskatoon, Saskatchewan, Canada; 7 School of Pharmacy, University of Waterloo, Kitchener, Ontario, Canada; 8 Faculty of Medicine, McGill University and Winslade Consultants Inc, Montreal, Quebec, Canada; 9 Scientist, C.T. Lamont Primary Health Care Research Centre, Élisabeth Bruyère Research Institute, 43 Bruyère Street, Ottawa, Ontario K1N 5C8, Canada

**Keywords:** Primary health care, Pharmacy, Pharmacists, Competencies, Scope of practice

## Abstract

**Background:**

Pharmacists have expanded their roles and responsibilities as a result of primary health care reform. There is currently no consensus on the core competencies for pharmacists working in these evolving practices. The aim of this study was to develop and validate competencies for pharmacists' effective performance in these roles, and in so doing, document the perceived contribution of pharmacists providing collaborative primary health care services.

**Methods:**

Using a modified Delphi process including assessing perception of the frequency and criticality of performing tasks, we validated competencies important to primary health care pharmacists practising across Canada.

**Results:**

Ten key informants contributed to competency drafting; thirty-three expert pharmacists replied to a second round survey. The final primary health care pharmacist competencies consisted of 34 elements and 153 sub-elements organized in seven CanMeds-based domains. Highest importance rankings were allocated to the domains of care provider and professional, followed by communicator and collaborator, with the lower importance rankings relatively equally distributed across the manager, advocate and scholar domains.

**Conclusions:**

Expert pharmacists working in primary health care estimated their most important responsibilities to be related to direct patient care. Competencies that underlie and are required for successful fulfillment of these patient care responsibilities, such as those related to communication, collaboration and professionalism were also highly ranked. These ranked competencies can be used to help pharmacists understand their potential roles in these evolving practices, to help other health care professionals learn about pharmacists' contributions to primary health care, to establish standards and performance indicators, and to prioritize supports and education to maximize effectiveness in this role.

## Background

The increasing burden of chronic conditions on patients, their families and communities, and the health system, is leading the developed world to investigate new approaches to caring for patients. Primary health care, as the first level of contact with the health system for many individuals, has been refocused to emphasize health promotion, illness prevention and chronic disease management [1]. To address the challenge of access to primary health care, interprofessional models are emerging. Pharmacists are playing a growing part in primary health care reform by fulfilling an increasing range of roles and responsibilities.

While pharmacists are well established in providing primary health care services in community pharmacy practice, their role and contribution to primary health care teams and their expanded roles in community pharmacy practice are being discovered as health care reforms unfold in many countries. In the majority of provinces in Canada, the scope of pharmacist practice is being expanded and regulations and reimbursement models are evolving to further support pharmacist provision of primary health care [2,3]. However, to ensure the optimal function of emerging interprofessional primary care teams, it is essential to clearly understand the scope and roles of the various professions participating in these teams [4]. For the profession of pharmacy in Canada, although entry-to-practice competencies exist, there are currently no competencies articulated that focus on the roles of pharmacists in the evolving primary health care field. The aim of this study was to develop and validate competencies for pharmacists' effective performance in these roles, and in so doing, document the perceived contribution of pharmacists providing collaborative primary health care services.

## Methods

We used a modified Delphi technique with initial expert development of a draft list of competencies followed by two online surveys and a teleconference to validate competencies important to primary health care pharmacists practising across Canada.

### Primary health care pharmacist competency framework development

Core primary health care competencies required for pharmacists were drafted based on review of innovative primary health care pharmacists' practices, existing entry-to-practice competencies and educational outcomes for Canadian pharmacists and key literature review [2,5-10]. The framework from the Association of Faculties of Pharmacy (AFPC) educational outcomes for Canadian pharmacists was selected in order to illustrate the differences in competencies expected of pharmacists who focus on provision of primary health care in community or primary health care team settings relative to the competencies required of newly graduated pharmacists who provide primary health care routinely as part of patient consultation and dispensing of medications [5]. This format also aligns with the CanMeds model, which is the Royal College of Physicians and Surgeons of Canada's framework that identifies the essential competencies required of physicians [11]. The CanMeds framework articulates seven roles and associated competencies required of physicians; medical expert (central role), communicator, manager, health advocate, scholar and professional. A number of professions and organizations in Canada have adopted the CanMeds format when defining competencies required for their professions, including the Canadian Patient Safety Institute [12] and the Canadian Association of Physician Assistants [13]. Adoption of this common format for the primary health care pharmacist competencies was meant to support understanding of the roles and responsibilities of pharmacists relative to the roles of other members of the primary health care team. Using this format, domain descriptions focused on identifying the emerging roles of pharmacists in primary health care environments. Competency elements were key features of the domains that provided a description of the core tasks, activities or responsibilities of the primary health care pharmacist (PHCP). The competency sub-elements provided detail of the competencies required of pharmacists to fulfill these primary health care roles.

### Key informant review

The proposed competencies were vetted by 10 key informants selected purposefully by the project team as pharmacists who were leaders in patient care, education, or research in the primary health care setting and provided representation from across Canada. They reviewed the proposed competencies for representativeness and relevance to pharmacy practice considering their role and experience in the primary health care setting, provided written comments and gave feedback in telephone interviews. Based on this review, draft competencies for primary health care pharmacists were prepared.

### Modified Delphi validation

The project team identified Canadian pharmacists with practices that focussed on providing direct patient care and follow up, who had a collaborative working relationship(s) with one or more physicians and who documented ongoing care in patient records (i.e. providing primary health care at an expert or proficient level) [14]. Such pharmacists were identified via review of innovative practices described in the Pharmacy Moving Forward project which identified and documented emerging innovative pharmacy practices and models across Canada [15] and review of members of the Ontario Pharmacist Family Health Team Listserv. Identified pharmacists were contacted by e-mail to both to obtain their consent to participate in the competency validation surveys, a teleconference call and to complete the surveys. To encourage participation, pharmacists were informed that a participant who had completed the surveys would be randomly selected to receive a $100 gift certificate.

The first of the on-line surveys functioned as a pilot to test both the clarity of the survey instructions and the thoroughness of the draft PHCP competency description. Open comments were reviewed to identify common concerns identified by the respondents. To further clarify concerns and revise the competencies, respondents who had partially or fully completed the questionnaire were invited to participate in a teleconference. Participants were presented with a brief summary of the ratings results of the first survey and asked to discuss the format of the survey, the rating scales, sub-element wording and applicability to individual's practice. Revisions were made to the original competency document and survey based on the feedback received from the first survey, the teleconference and review and comments by research team members. In addition, at the time of the review, AFPC had released a final version of the educational outcomes that had included some revisions to the original document used for developing the PHCP competencies. The research team reviewed all of these comments and documents and made specific revisions to the PHCP competency document to clarify wording and reduce the number of sub-elements. The survey rating scales were also adjusted for the criticality question to make sure the relevance of competencies to practice, the profession and society were easier to comprehend. The revised survey was then re-administered to the same group of pharmacists to obtain the final validation ratings of the PHCP competencies.

The surveys requested pharmacists rate each draft competency sub-element on a six-point Likert scale, identifying first how often they performed the sub-element in practice and second how critical they believed competent performance of the sub-element was to achievement of the patient's desired health outcomes [16]. Open comment boxes were used to invite respondents to comment on clarity and missed items.

For each sub-element a mean frequency and mean criticality rating were calculated by considering all respondents' ratings. For partially completed surveys, mean criticality and frequency values were imputed and sensitivity analysis was done based on the pharmacists with complete data. Using methodologies established by Kane et al., [16] an initial importance weighting was calculated for each sub-element by multiplying the mean frequency and criticality values. This importance weighting was then adjusted to ensure that criticality and frequency contributed equally to the resulting importance weighting. This adjustment was necessary to accommodate for the greater variability inherent to frequency weightings relative to criticality weightings, since this greater variability lead to an undesirable higher contribution of frequency when simple multiplication was used.

To calculate importance weightings for elements, the frequency and criticality ratings for sub-elements that belonged to the same element were aggregated [16]. Values of elements which belong to the same domain were then aggregated to create domain level frequency and criticality ratings. Adjusted importance weights were then calculated for each element and domain using the same adjustment procedure as for the sub-elements to ensure equal contribution of criticality and frequency.

This study was approved by the Bruyère Continuing Care Research Ethics Board.

## Results

Both the first round and second round surveys were sent to 87 pharmacists who had been identified as providing primary health care at the proficient or expert level. In the first round 16 pharmacists responded partially or completely (response rate = 18%) and in the second round of the survey, 33 pharmacists partially or fully completed the survey (response rate = 38%). Table 1 provides the demographic data on key informants (N = 10), teleconference participants (N = 4) and the 21 of 33 second round survey respondents who provided demographic information (N = 21). Only complete responses were included in this analysis so there are no missing values from these demographic characteristics.

**Table 1:** Participant demographics

	Key informants N = 10 n (%)	Teleconference Participants N = 4 n (%)	Final Survey N = 21* n (%)
**Gender**			
**Female**	8 (80%)	2 (50%)	13 (62%)
**Male**	2 (20%)	2 (50%)	8 (38%)
**Age**			
**20 - 29 years**	0	0	0
**30 - 39 years**	2 (20%)	1 (25%)	8 (38%)
**40 - 49 years**	6 (60%)	2 (50%)	8 (38%)
**50 - 59 years**	2 (20%)	1 (25%)	2 (9.5%)
**60 - 69 years**	0	0	2 (9.5%)
**70 years and above**	0	0	1 (5%)
**Practice Type^**			
**Community pharmacy**	1 (10%)	1 (25%)	5 (24%)
**Ambulatory Clinic**	1 (10%)	0	4 (19%)
**Consulting to family practice**	0	0	3 (14%)
**Integrated into family practice**	3 (30%)	3 (75%)	12 (57%)
**Other (e.g. academia, health care organization)**	8 (80%)	0	2 (10%)

* 21 of the 33 respondents completed the demographics section in the final survey.

^ More than one practice site is indicated for some participants.

Consistent with trends in pharmacy practice, the majority of participants were female [17]. Given that participants had to be proficient or expert, it was also anticipated that participants would have a number of years of practice experience and Table 1 confirms this expectation. While key informants were heavily represented by those integrated into family practice or in academia, respondents to the final survey represented a wide range of primary health care environments, including community pharmacy.

The revised PHCP competencies that formed the basis of the validation (second) survey had seven domains, 34 elements and 153 sub-elements. The seven domains and role definitions are described below.

**As Medication Therapy Experts, primary health care pharmacists (PHCPs) integrate knowledge, skills and professional attitudes to effectively contribute to improved quality of drug therapy through the provision of patient-centred care and in collaboration with health care providers**.

In functioning as a Medication Therapy Expert, PHCPs fulfill roles relating to care and services for individual patients as well as roles emphasizing the responsibilities of pharmacists to populations of patients, and to their communities.

Role Definitions:

**Advocate**: Primary health care pharmacists use their expertise and influence to advance the health and well-being of individual patients, communities and populations.

**Care Provider**: Primary health care pharmacists use their knowledge and skills to provide pharmaceutical care and to facilitate management of patient's medication and overall health needs.

**Collaborator**: Primary health care pharmacists work collaboratively with patients, family physicians, and other primary health care professionals and in teams to provide effective, quality health care and to fulfill their professional obligations to the community and society at large.

**Communicator: **Primary health care pharmacists communicate with diverse audiences, using a variety of strategies that take into account the situation, intended outcomes of the communication and the target audience.

**Manager: **Primary health care pharmacists use management skills in their daily practice to optimize the care of patients, and to make efficient use of health resources.

**Professional**: Primary health care pharmacists honour their roles as self-regulated professionals through both individual patient care and fulfillment of their professional obligations to the community and society at large.

**Scholar: **Primary health care pharmacists have and can apply the core information required to be a medication therapy expert, and are able to master, generate, interpret and disseminate pharmaceutical knowledge.

Table 2 provides an example of a competency domain, description, one of its' elements and its' related sub-elements (for the role of patient care). Additional file 1 provides the complete PHCP competencies.

**Table 2:** Example of competency domain, element, and sub-elements

***Domain***	*2. As Care Providers, primary health care pharmacists use their knowledge and skills to provide pharmaceutical care and to facilitate management of patient's medication and overall health needs*.
***Description***	*Primary health care pharmacists possess the core knowledge, skills and attitudes required to be able to:* *i. manage patients:* • *who require the pharmacist's participation in their care;* • *who are willing and able to accept the responsibilities required by this care;* • *with common and uncommon medication-therapy problems or complex medication-related needs; and identify patients with highly complex medication-related needs*. • *who require urgent care and provide basic first aid and CPR;* *ii. are able to acquire the knowledge and skills required to manage patients with highly complex medication-related needs*. *iii. provide care in accordance with accepted frameworks that expand the pharmacist's scope of practice (e.g. medical directives);* *iv. recommend appropriate sources of support for patients experiencing common difficulties in daily living;* *v. advise patients on common, current health promotion campaigns;* *vi. are able to refer patients for the management of medication therapy needs that fall beyond their individual scope of practice;* ***vii*. ***are able to triage patients to other primary health care providers*.
***Element***	*Elicit and complete an assessment of required information to determine the patient's medication-related and relevant health needs*.
***Sub-element***	*elicit the reason(s) for the patient's visit or referral to the pharmacist;*
***Sub-element***	*obtain and evaluate relevant history from the patient, his/her chart, caregivers and other health care professionals (e.g. medication experience, medication history, current medication record, past and current medical history, allergies, immunizations, social drug use, previous adverse reactions, etc);*
***Sub-element***	*order and/or retrieve and assess relevant lab tests and diagnostic tests;*
***Sub-element***	*perform and interpret findings of relevant physical assessment;*
***Sub-element***	*complete an assessment of the patient's ability to take/use/administer his/her medications*.

Outlined below is the final wording used in the validation survey for pharmacists to rate the frequency and criticality of performance of each sub-element in the final PHCP competencies.

Frequency question

As your practice develops in the next 5 years, how frequently do you see yourself performing each of the listed tasks (assuming you are working full-time)?

6 - continuously (i.e. hourly or more)

5 - between two and six times per day (but not hourly)

4 - once per day (i.e. five times/week)

3 - between one and four times/week (but not daily)

2 - between one and three times per month (but not weekly)

1 - less than once per month

Criticality question

Considering each time that the competency unit should be performed, what risk (immediate or long term) would it cause patients, either directly or by affecting access to health services, if you did not perform the task competently?

6 - life threatening

5 - serious consequences

4 - worsens situation

3 - prevents improvement in situation

2 - causes inconvenience

1 - no impact

Competency elements directly related to provision of patient care ranked highest including 'elicit and complete an assessment of required information to determine the patient's medication-related and relevant health needs' (ranked 1) and 'assess if a patient's medication-related needs are being met' (ranked 2). Competencies that ranked lowest were less directly linked to provision of patient care including 'participate in practice research' (ranked 33) and 'formally educate diverse audiences regarding medications and appropriate medication use, health promotion or self-management' (ranked 34). The adjusted importance rankings for the competency elements are shown in Table 3. Complete frequency and criticality ratings and importance rankings for all sub-elements are provided in online Additional file 2.

**Table 3:** Importance rankings (rank adjusted) for competency elements

Domain	Competency Element	F	C	I	Rank
Advocate	Promote the health of individual patients	4.63	3.80	3.40%	16
	Promote the health of patients and patient groups within their communities	2.17	2.99	0.88%	30
	Support the role of pharmacists in the primary health care system	2.92	3.24	1.45%	27
Health care Provider	Develop and maintain professional, collaborative relationships required for patient care	5.52	4.03	4.69%	6
	Elicit and complete an assessment of required information to determine the patient's medication-related and relevant health needs	5.24	4.46	5.70%	1
	Assess if a patient's medication-related needs are being met	5.20	4.39	5.44%	2
	Determine if a patient has health needs that require management	4.06	4.72	5.09%	4
	Refer patients for management of priority health and wellness needs that fall beyond the scope of practice of pharmacists	3.55	3.91	2.79%	18
	Develop a shared plan of care that addresses a patient's medication-therapy problems and priority health needs	5.11	3.79	3.37%	13
	Implement the care plan	4.79	3.68	3.25%	17
	Elicit clinical and/or lab evidence of patient outcomes	4.71	4.49	5.21%	3
	Assess and manage patients' new medication-related needs	4.70	4.10	4.16%	9
	Document their patient care activities	5.08	4.22	4.82%	5
Collaborator	Function as members of teams	4.53	3.16	2.81%	23
	Work collaboratively with the patient and his/her health care professionals to provide care and services that facilitate management of the patient's health needs	4.32	4.01	3.41%	14
Communicator	Communicate non-verbally and verbally with others	5.66	3.68	4.10%	12
	Communicate in writing	5.28	3.16	3.28%	21
	Present information	2.16	2.68	1.14%	31
	Use communication technology	3.68	2.97	2.15%	26
	Communicate effectively in special high-risk situations and address challenging communication issues	3.88	4.39	3.35%	10
Manager	Manage their personal practice	4.74	3.47	3.23%	19
	Support the sustainability of their practice	2.70	2.99	1.58%	28
	Participate in the development of policies and procedures supportive of the safe and effective use of medications and the provision of quality primary health care	3.33	3.21	2.10%	24
	Recognize the occurrence of errors and unsafe practices and respond effectively to mitigate harm to the patient, ensure disclosure, and prevent recurrence	2.11	4.55	1.88%	22
	Participate in quality assurance and improvement programs	2.20	3.24	1.40%	29
Professional	Demonstrate professionalism throughout patient encounters	5.78	3.68	4.18%	11
	Practice in an ethical manner which assures primary accountability to the patient	5.18	3.98	4.05%	8
	Practice in a manner demonstrating professional accountability	5.67	3.95	4.40%	7
	Ensure their personal competence to fulfil the evolving primary health care pharmacist's role	2.83	3.40	1.90%	25
	Support the profession and its evolving role in the primary health care system	1.89	2.83	1.05%	32
Scholar	Demonstrate a thorough understanding of the fundamental knowledge required of pharmacists by applying this knowledge in daily practice	4.49	3.92	3.45%	15
	Provide drug information and recommendations regarding medications and appropriate medication use for uptake and implementation into practice	4.12	3.52	2.85%	20
	Formally educate diverse audiences regarding medications and appropriate medication use, health promotion or self-management	1.54	2.78	0.52%	34
	Participate in practice research	1.54	2.81	0.54%	33

Adjusted percentage weights and ranks assigned to competency elements using I = CF where C and F contributed equally to Importance (weight)

**Figure 1:**
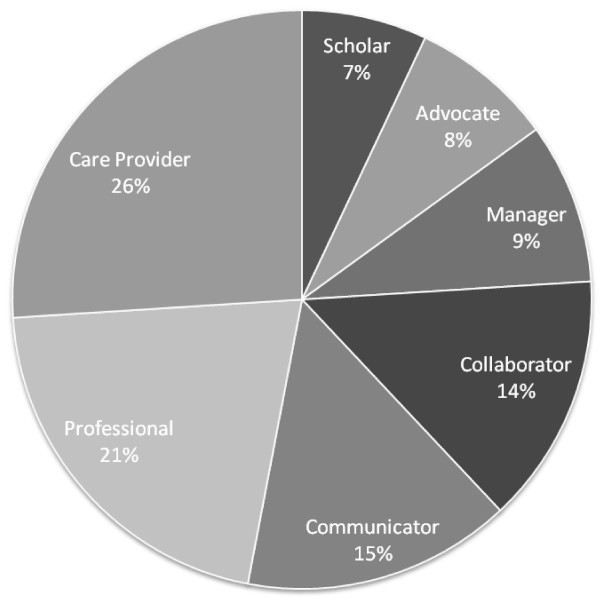
**Adjusted importance weight for the competency domains**.

Adjusted importance weight for the seven competency domains is shown in Figure 1. The results indicate that PHCPs fulfill a variety of roles related to the provision of patient care, collaboration with health care providers and meeting health system needs. The 'care provider' domain held the highest importance (26%) following closely by 'professional' (21%). "Health advocate", 'scholar' and 'manager' domains had the lowest importance weightings, although when combined (24%) still represent a significant contribution to PHCP roles.

## Discussion

A comprehensive set of competencies required of pharmacists to provide primary health care was developed and validated by practising primary health care pharmacists. Despite the increasing emphasis on primary health care and the transition to collaborative patient care teams, to our knowledge, this is the first literature that defines the primary health care roles and responsibilities that pharmacists can fulfill within these teams.

The PHCP competencies expand on the newly developed national educational outcomes for pharmacy graduates [5] by describing the competencies required of pharmacists who focus their practice on providing primary health care in a range of practice settings. The inclusion of specific primary care-related competencies in, for example, advocacy and management provide additional guidance as to how pharmacists not only provide primary health care to individual patients, but also how they fulfill primary health care responsibilities to communities and the health care system. The PHCP competencies are also consistent with Model Standards of Practice for Canadian Pharmacists [9], although the format of the PHCP competencies was selected specifically to align with the CanMeds format that is being adopted by professions across Canada [11].

Consistent with expectations, the competencies most directly related to patient care were rated the most important by practising pharmacists. The top five ranked competency elements focused on the pharmacist's expertise in identifying and managing medication therapy problems that affect patient's overall health outcomes. Competencies that underlie and are required for successful fulfillment of these patient care responsibilities, such as those related to communication, collaboration, and professionalism, were also highly ranked. Consistent with other professions in Canada, the competencies related to management, health advocacy, and scholarship were rated lower in both frequency and criticality [18,19]. These lower ratings could reflect a lower relevance of these activities relative to the direct patient care activities which are the main focus of most practicing pharmacists not in full time administration or teaching and research. Pharmacists may have provided lower ratings to those less frequently performed competencies which require a system-level focus as their effect is less immediately obvious compared to recalling direct patient care which can often be quantified in numbers seen, and impact assessed by immediate adverse events avoided or questions answered. Nonetheless, these non-direct patient care competencies still represented almost one quarter of the overall role importance for these primary health care pharmacists. As with other health professionals, further study is needed to determine the reason non direct patient care competencies receive lower ratings for criticality despite the professions' identification of these as fundamental to the practice of pharmacy [2]. It might be helpful to educators to determine if pharmacists have similar challenges to physicians in interpreting the meaning and relevance of competencies such as health advocacy and management [20,21].

As pharmacists have increasingly expanded their role to function as primary health care providers and the primary health care system continues to evolve, gaps in knowledge, and skills have been identified that indicate a need for bridge or specialty education [22-24]. The articulation of competencies and prioritization of pharmacist roles in primary health care have been helpful in informing the development of educational and continuing professional development programs such as the ADapting Pharmacist's skills and Approaches to maximize Patient's drug Therapy effectiveness (ADAPT) program for primary health care pharmacists [25].

Strengths of this work include the broad range of resources consulted during the initial development of the draft PHCP competencies, the inclusion of pharmacists who provide primary health care in a range of practice sites, the sampling of pharmacists across Canada and the methodologically rigorous approach to rating of the importance of the PHCP competencies. Rather than relying on group consensus or participant's use of their personal perspectives of the importance of the varying roles, a standardized rating scale was developed, piloted and refined prior to final use. Although the origins of Kane's criticality and frequency ratings methodology was for use in the development of entry-to-practice examinations, the methodology provided a robust approach to standardizing participant's approaches to the rating of importance. Modifications in the rating scales and questions were required to recognize that some primary health care activities are provided on a relatively continuous basis in the background of performance of professional tasks. For example, the most frequent category was changed to 'continuously' rather than relying on a discrete number of times per day (e.g. 7 or more times per day). For the criticality rating, the question asked also required modification to specify that responsibilities could either directly or indirectly affect patient's outcomes. This allowed pharmacists flexibility in considering the impact of competencies related to, for example, management or advocacy on patient outcomes through mechanisms such as ensuring access to timely and competent care and services.

There are some limitations to this study. Response rates were low and the PHCP competencies were, therefore, validated by a limited number of primary health care pharmacists. Further work should be done to determine if these results are relevant to a greater number of pharmacists who provide primary health care. However, relying on those pharmacists with the most expertise should have provided a comprehensive set of competencies to identify the greatest range of potential primary health care roles for pharmacists. A second limitation is that the competency validation was based on the roles and responsibilities of pharmacists as projected for the next five years. There may be roles and responsibilities that will continue to evolve that necessitate re-examination of these PHCP competencies over time.

Future work should consider wider validation of these PHCP competencies through consultation with a larger group of practising pharmacists and the consideration of perspectives of stakeholders such as patients, other health care professionals working in primary health care teams and provincial governments. This would allow a broader perspective on the importance of these roles of primary health care pharmacists. In addition to this further validation of perceived roles, empirical evaluation of the frequency, criticality and impact of pharmacists' fulfillment of these roles is necessary. New techniques that have been validated for evaluating the quality of pharmacist's daily performance would be useful in these studies [26].

## Conclusions

In summary, competencies for primary health care pharmacists have been developed and validated that clarify the roles that pharmacists can fulfill and outline the contributions that pharmacists could make to an effective primary health care system. Direct patient care with emphasis on managing patients' medication-related needs remains the clear focus of primary health care pharmacists, with recognition that fulfillment of this responsibility requires communication, collaboration and professionalism. Less highly rated are roles related to management, health advocacy and scholarship and further work is necessary to determine the reason for these lower ratings. This validated list of competencies for pharmacists practicing in primary health care should be of use to practitioners, policy-makers, and educators and is an important first step to developing measures for assessing the performance and impact of pharmacists' provision of primary health care services.

## Abbreviations

(PHCP): Primary Health Care Pharmacist; (ADAPT): ADapting Pharmacist's skills and Approaches to maximize Patient's drug Therapy effectiveness

## Competing interests

The authors declare that they have no competing interests.

## Authors' contributions

NK developed the competencies, participated in study design/methodology and analysis and drafted the manuscript. BF was the study principal investigator, directed the study design/methodology, participated in analysis and made critical revisions to the manuscript. NWard contributed to study design/methodology, participated in data acquisition and analysis and writing of the manuscript. SJ participated in analysis and writing of the manuscript. AG participated in data acquisition and analysis and editing of the manuscript. TW participated in statistical data analysis and editing of the manuscript. LD contributed to study design/methodology and analysis and editing of the manuscript. DJ and NWaite participated in data analysis and editing of the manuscript. NWinslade contributed to competency development, participated in study design/methodology and analysis and made critical revisions to the manuscript. All authors have read and approved the final manuscript.

## Pre-publication history

The pre-publication history for this paper can be accessed here:

http://www.biomedcentral.com/1471-2296/13/27/prepub

## Supplementary Material

Additional file 1 **Primary Health Care Competencies**. Primary Health Care Pharmacist Competencies. This document provides role definitions, competencies and competency sub-elements for the Primary Health Care Pharmacist competency framework. Click here for file

Additional file 2 **Importance Rankings (rank adjusted) for Competency Sub-elements.** Importance Rankings (rank adjusted) for Competency Sub-elements. The document provides importance ratings for all primary health care competency elements and sub-elements. Click here for file
