# Supplementary material for: Pharmacist provision of primary health care: a modified Delphi validation of pharmacists' competencies
Source: BMC Fam Pract. 2012 Mar 28;13:27. doi: 10.1186/1471-2296-13-27 (PMC3372430; doi:10.1186/1471-2296-13-27)
Supplement: Additional file 1 — Primary Health Care Competencies. Primary Health Care Pharmacist Competencies. This document provides role definitions, competencies and competency sub-elements for the Primary Health Care Pharmacist competency framework. [file 1471-2296-13-27-S1.PDF]

Appendix 1

Primary Health Care Pharmacist Competencies

 Contact natalie.kennie@utoronto.ca.

Primary Health Care Pharmacist Competencies

As Medication Therapy Experts, primary health care pharmacists (PHCPs) integrate knowledge, skills and professional attitudes to effectively contribute to improved quality of drug therapy through the provision of patient-centred care and in collaboration with health care providers. In functioning as a Medication Therapy Expert, PHCPs fulfill roles relating to care and services for individual patients as well as roles emphasizing the responsibilities of pharmacists to populations of patients, and to their communities. Roles definitions are presented in alphabetical order.

Role Definitions:

Advocate: Primary health care pharmacists use their expertise and influence to advance the health and well-being of individual patients, communities and populations.

Care Provider: Primary health care pharmacists use their knowledge and skills to provide pharmaceutical care and to facilitate management of patient's medication and overall health needs.

Collaborator: Primary health care pharmacists work collaboratively with patients, family physicians, and other primary health care professionals and in teams to provide effective, quality health care and to fulfill their professional obligations to the community and society at large.

Communicator: Primary health care pharmacists communicate with diverse audiences, using a variety of strategies that take into account the situation, intended outcomes of the communication and the target audience.

Manager: Primary health care pharmacists use management skills in their daily practice to optimize the care of patients, and to make efficient use of health resources.

Professional: Primary health care pharmacists honour their roles as self-regulated professionals through both individual patient care and fulfillment of their professional obligations to the community and society at large.

Scholar: Primary health care pharmacists have and can apply the core information required to be a medication therapy expert, and are able to master, generate, interpret and disseminate pharmaceutical knowledge.

Contact natalie.kennie@utoronto.ca.

Advocate

As ***Advocates*** primary health care pharmacists use their expertise and influence to advance the health and well-being of individual patients, communities, and populations.

Description:
PHCPs advocate on behalf of individual patients, local patient groups and the profession of pharmacy, at an individual, organizational / institutional, and government level. In addition, PHCPs play an active role in advancing the health and well-being of patients, by participating in health promotion and disease prevention activities consistent with the role of the pharmacist and in collaboration with other primary health care professionals.

As **Advocates** primary health care pharmacists:[1-4]

1.1 Promote the health of individual patients.

1.1.1 facilitate patient's interaction with the health care system through advice, education and/or guidance; and
1.1.2 support patient's access to required health services by representing or speaking on behalf of patients.

1.2 Promote the health of patients and patient groups within their communities.

1.2.1 determine healthcare needs of local patients and patient groups and implement programs/services to address or support those needs.
1.2.2 *participate*[1] in health promotion activities, public health campaigns and patient safety initiatives that are directed at disease prevention, risk factor reduction and/or harm minimization;
1.2.3 undertake relevant public health screening processes for early disease detection; and
1.2.4 assist in planning and implementing public health promotion education and awareness raising campaigns with other health professionals.

1.3 Support the role of pharmacists[2] in the primary health care system.

1.3.1 promote the impact of the pharmacist on patient outcomes; and
1.3.2 promote the role of pharmacists in the development and implementation of health procedures, policies and services.

---

[1] Primary health care pharmacists are expected to participate and assist in the development of health promotion and public health strategies/programs, rather than independently lead these initiatives as this would require attainment of additional competencies for leadership or mastery.

[2] For the purpose of the primary health care pharmacist competencies, this document focuses on professional competencies that relate directly to improving the care of patients, collaboration with health care providers and contributions to the health care system. Professional competencies related to advancing the role of pharmacists in general are not included.

Contact natalie.kennie@utoronto.ca.

Care Provider

As ***Care Providers,*** primary health care pharmacists use their knowledge and skills to provide pharmaceutical care and to facilitate management of patient's medication and overall health needs.

Description:
Primary health care pharmacists possess the core knowledge, skills and attitudes required to be able to:

i. manage patients:
   - who require the pharmacist's participation in their care;
   - who are willing and able to accept the responsibilities required by this care;
   - with common and uncommon medication-therapy problems or complex medication-related needs; and identify patients with highly complex medication-related needs.
   - who require urgent care[3] and provide basic first aid and CPR;

ii. are able to acquire the knowledge and skills required to manage patients with highly complex medication-related needs.

iii. provide care in accordance with accepted frameworks that expand the pharmacist's scope of practice (e.g. medical directives);

iv. recommend appropriate sources of support[4] for patients experiencing common difficulties in daily living[5];

v. advise patients on common, current health promotion campaigns;

vi. are able to refer patients for the management of medication therapy needs that fall beyond their individual scope of practice;

vii. are able to triage patients to other primary health care providers.[6]

As **Care Providers,** primary health care pharmacists:[1,5-9]

A. Assess patients

2.1 Develop and maintain professional, collaborative relationships required for patient care.

2.1.1 establish and maintain a professional, caring practice environment;
2.1.2 demonstrate that the patient's goals are the priority;
2.1.3 determine when it is ethically and professionally appropriate to involve caregivers; and
2.1.4 acknowledge and respect the roles and responsibilities of the pharmacist, the patient and/or caregivers, and the patient's other health care professionals.

2.2 Elicit and complete an assessment of required information to determine the patient's medication-related and relevant health needs.

2.2.1 elicit the reason(s) for the patient's visit or referral to the pharmacist;
2.2.2 obtain and evaluate relevant history from the patient, his/her chart, caregivers and other health care professionals (e.g. medication experience, medication history, current medication record, past and current medical history, allergies, immunizations, social drug use, previous adverse reactions, etc);
2.2.3 order and/or retrieve and assess relevant lab tests and diagnostic tests;*
2.2.4 perform* and interpret findings of relevant physical assessment; and;
2.2.5 complete an assessment of the patient's ability to take / use / administer his/her medications.

2.3 Assess if a patient's medication-related needs are being met.

2.3.1 evaluate the appropriateness, safety and effectiveness of a patient's medications with

---

[3] Urgent medication therapy needs are those that require urgent care by the pharmacist or urgent referral to primary care providers (e.g. via ambulance or referral to ER)
[4] Primary health care pharmacists should have knowledge of specific community resources and the process of accessing these resources.
[5] Difficulties with, for example, transportation, activities of daily living, emotional, spiritual needs.
[6] Including, for example, signs and symptoms of diabetes mellitus, hypertension, arthritis, stroke, cardiac disease. Primary care pharmacists should be able to identify patients requiring assessment and referral to other health care providers.

 Contact natalie.kennie@utoronto.ca.

consideration of the patient's characteristics, values/preferences, conditions, functional capabilities, other medications and access to health care / monitoring;

2.3.2 determine whether a patient is appropriately managing his/her therapy, including appropriate administration and adherence in particular for chronic disease management;

2.3.3 determine whether a patient's medications are achieving the desired goals including consideration of efficacy and adverse effects;

2.3.4 determine whether a patient requires medication assessment and reconciliation;

2.3.5 identify a patient's medication-related needs as specific medication-therapy problems, and

2.3.6 determine if a patient requires additional care or services consistent with established collaborative practice agreements.

2.4 Determine if a patient has health needs that require management.

2.4.1 recognize signs, symptoms and risk factors that relate to medical or health problems that fall into the scope of practice of other health care professionals;

2.4.2 recognize signs and symptoms associated with medical emergencies; and

2.4.3 recognize problems with activities of daily living important to the patient's well- being.

B. Plan Care

2.5 Refer patients for management of priority health and wellness needs that fall beyond the scope of practice of pharmacists.

2.6 Develop a shared plan of care that addresses a patient's medication-therapy problems and priority health needs.

2.6.1 prioritize a patient's medication-related needs;

2.6.2 establish goals of drug therapy with the patient (desired endpoints, target values and timeframes for medication therapies);

2.6.3 assess alternative strategies and negotiate the therapeutic option best suited to the patient in collaboration with the patient and other health care professionals;

2.6.4 integrate the recommended therapeutic options for a patient's medication-related needs into a co ordinated plan;

2.6.5 determine monitoring parameters for desired therapeutic endpoints and potential adverse effect, specifying target values and start, frequency and end time-points for monitoring;

2.6.6 decide specific actions to be taken by the pharmacist as necessary for the management of medication-related needs, specifically determining whether it is appropriate to:

- dispense a medication according to a new prescription;
- dispense an authorized refill of a medication;
- authorize an extension of refills of a medication;*
- modify a patient's medications;*
- recommend changes in medications;
- prescribe medications or therapies;*
- administer a medication*, and/or;
- refer to other health care professionals for assessment and management;

2.6.7 determine if a patient requires information to facilitate his/her management of needs related to activities of daily living, health promotion or well-being; and

2.6.8 negotiate the care plan responsibilities of the pharmacist and patient, and when other health care professionals should be contacted.

2.7 *Implement the care plan.*

2.7.1 undertake specific actions as specified in the care plan (e.g. prescribing, ordering labs*);

2.7.2 educate a patient regarding the care plan to facilitate understanding and adherence;

2.7.3 facilitate the continuity of care through referral and communication with relevant care providers;

2.7.4 convey information on maintaining and promoting health and self-management;

2.7.5 convey information about available social support services to assist with daily living; and;

2.7.6 schedule required follow-up in accordance with a patient care plan.

C. Follow-up and Evaluate

Contact natalie.kennie@utoronto.ca.

2.8 Elicit clinical and / or lab evidence of patient outcomes.<br>2.8.1 evaluate the efficacy of the care plan relative to the desired goals;<br>2.8.2 evaluate the safety of the care plan including the presence of adverse drug reactions or effects;<br>2.8.3 recognize, disclose and manage adverse drug events;<br>2.8.4 identify any medication errors or close calls[7]; and<br>2.8.5 determine changes in pharmacotherapy that are required.
2.9 Assess and manage patients' new medication-related needs[8].
**D. Document**
2.10 Document their patient care activities.<br>2.10.1 document in a timely, retrievable, usable manner;<br>2.10.2 maintain, clear, accurate and appropriate records for all patient encounters;<br>2.10.3 document their decisions/actions, supporting patient and related information and their interpretation of this information;<br>2.10.4 document communication with other health care providers and health/social agencies;<br>2.10.5 document the reporting of adverse events.

[7] A close call is defined as an event with the potential for harm that did not result in harm because it did not reach the patient sue to timely intervention or good fortune.
[8] Reassess patient at regular intervals to identify new medication-related needs. Go back to assessing patients section.

Contact natalie.kennie@utoronto.ca.

Collaborator

As ***Collaborators*** primary health care pharmacists work with patients, family physicians and other health care professionals and teams to support access to and provision of effective, quality health care and to fulfill their professional obligations to the community and society at large.

Description:
PHCPs effectively collaborate with the patient and the full range of health care professionals that make up the patient's health care team to support access to and provision of quality health care**.** Effective collaborative relationships are formed with family physicians and other health care professionals in primary health care, including those within the pharmacy profession. Care roles are negotiated by health care professionals involved in the care of the patient. Collaboration in primary health care may occur within and across patient care sites.

As **Collaborators** primary health care pharmacists:[1,10,11]

3.1. Function as members of teams.

3.1.1 clarify roles, responsibilities and expertise of other professions, identifying overlaps and gaps;
3.1.2 recognize and respect the roles, responsibilities and competence of other professions;
3.1.3 accept leadership roles where appropriate;
3.1.4 actively make their expertise available to others and willingly agree to share relevant information, using language that can be understood by all;
3.1.5 make their points of view known, listen to and respect the opinions of others, defend points of view if necessary;
3.1.6 contribute to planning, organizing and performing the patient care to be provided and evaluating the results;
3.1.7 respect the rules established by the group;
3.1.8 help maintain a healthy work environment and assist with conflict management;
3.1.9 support continued efforts of the group by providing positive feedback, including evidence of progress and impact;
3.1.10 facilitate discussion and interaction among team members;
3.1.11 participate and be respectful of all members' participation in collaborative decision-making; and
3.1.12 adapt their roles in teams and networks of care to the circumstances and requirements.

3.2 Work collaboratively with the patient and his/her health care professionals to provide care and services that facilitate management of the patient's health needs.

3.2.1 develop and maintain effective collaborative working relationships with a network of local health care professionals including the family physicians and those within the pharmacy profession;
3.2.2 ensure that the care and services that the pharmacist accepts to provide patients is consistent with laws /regulations relevant to collaborative care;
3.2.3 ensure their attainment and maintenance of training / certification / credentials required to provide collaborative care or to fulfill medical directives / delegation;
3.2.4 ensure legality of collaborative practice agreements / medical directives / delegation agreements;
3.2.5 plan the provision of care in a coordinated fashion;
3.2.6 provide agreed upon care and services;
3.2.7 communicate with other health care providers in a collaborative, responsive and responsible manner.
3.2.8 seek out, integrate and value, as a partner, the input and the engagement of the patient//family/ community and health care team in designing and implementing care/ services; and
3.2.9 identify and act on sub-optimal care issues, safety issues, priorities and adverse events in the context of team practice.

 Contact natalie.kennie@utoronto.ca.

Communicator

As ***Communicators*** primary health care pharmacists communicate effectively with diverse audiences, using a variety of strategies that take into account the situation, intended outcomes of the communication and the target audience.

Description:
PHCPs communicate effectively with patients to whom they are providing care and other health care professionals in both individual and group settings to foster effective patient care and interprofessional collaboration.

As **Communicators** primary health care pharmacists:[1,10,11]

4.1. Communicate non-verbally and verbally with others.

4.1.1 demonstrate active listening skills and respond appropriately;
4.1.2 exhibit empathy, tact and respect in their dealings with others;
4.1.3 recognize and respect cultural diversity and health literacy needs;
4.1.4 when speaking, use organized processes and appropriate, precise expressions and vocabulary;
4.1.5 tailor the content of their communication to specific contexts and audiences;
4.1.6 adapt their communication techniques to facilitate efficient and effective clinical encounters; and
4.1.7 convey information in a way that is understandable, and that encourages discussion and participation in decision-making.

4.2. Communicate in writing.

4.2.1 write clearly, and efficiently using organized processes and appropriate vocabulary;
4.2.2 correctly apply the rules of syntax, grammar and punctuation; and
4.2.3 tailor the content of their arguments to specific contexts and target audiences.

4.3. Present information.

4.3.1 appear comfortable, engage the audience, use appropriate tone and pace, and use nonverbal language appropriately;
4.3.2 organize presentation and sets and adheres to appropriate time limits; and
4.3.3 respond to and manage interaction with the audience;

4.4. Use communication technology.

4.4.1 use effective communication skills regardless of the media employed including effective use of visual and educational aids; and
4.4.2 effectively use information and communication technology to improve interprofessional patient-centred care.

4.5 Communicate effectively in special high-risk situations and address challenging communication issues.

4.5.1 recognize and effectively manage patient barriers to communication and understanding (e.g. cognition, mental illness, cultural, language, socioeconomic status, hearing and sight impairment, etc);
4.5.2 engage patients or substitute decision-makers in a discussion of risks and benefits of treatments and to obtain informed consent;
4.5.3 communicate to others the urgency of a clinical situation;
4.5.4 employ appropriate communication approaches in high-risk situations, such as in clinical crises, emotional or distressing situations, and conflict; and
4.5.5 use appropriate communication approaches to provide safe transfers, transitions of care and consultations among health care providers, within and between sites of care.
.

 Contact natalie.kennie@utoronto.ca.

Manager

As ***Managers*** primary health care pharmacists organize their practice to optimize the care of patients, support access to healthcare services and make efficient use of health resources.

Description:
Within their individual and collaborative practices, in the community and/or in health care organizations, PHCPs organize their practice to optimize the care of patients, support access to healthcare services and to make efficient use of health resources. They participate in making informed decisions about the safe and effective use of medication, allocation of resources and development of patient care services, considering the principles of primary health care, human and financial resources management, and common medication-related problems in primary care that lead to hospital admission, emergency room visits, morbidity and mortality.

As **Managers** primary health care pharmacists:[1,11,12]

5.1 *Manage their personal practice.*
- 5.1.1 manage their time to balance patient care, workflow and practice requirements; and
- 5.1.2 adapt their practice to fulfill evolving professional roles.

5.2 *Support the sustainability of their practice*
- 5.2.1 appropriately record workload and, where non-salaried, bill for patient care and professional services;
- 5.2.2 adapt their practice, providing new or emerging services as consistent with patient need and/or management's practice change plans;
- 5.2.3 incorporate the use of technologies to improve efficiency and effectiveness of their practice; and
- 5.2.4 ensure patient and team awareness of documented impact/value of their care and professional services.

5.3. *Participate in the development of policies and procedures supportive of the safe and effective use of medications and the provision of quality primary health care.*
- 5.3.1 develop and practice according to policies and procedures that support improved access to health services (e.g. collaborative practice agreements, medical directives);
- 5.3.2 develop and adhere to delegation and workflow policies that ensure efficient use of human resources; and
- 5.3.3 develop and incorporate processes supportive of patient self-management into their practice.

5.4 Recognize the occurrence of errors and unsafe practices and respond effectively to mitigate harm to the patient, ensure disclosure, and prevent recurrence.
- 5.4.1 recognize, disclose, manage and report errors, incidents and unsafe practices; and
- 5.4.2 participate in timely event analysis, reflective practice, and planning for the prevention of recurrence.

5.5 Participate in quality assurance and improvement programs.
- 5.5.1 ensure the care they provide is timely, cost effective and results in positive impact on care processes and/or patient outcome; and
- 5.5.2 participate[9] in planning for implementation and evaluation of services/programs to meet patient's needs and/or improve the quality of care provided, including strategies to identify and overcome barriers, and to capitalize on facilitators.

[9] Primary health care pharmacists are expected to participate and assist planning and implementation of services, rather than independently lead these initiatives as this would require attainment of additional competencies for leadership or mastery.

 Contact natalie.kennie@utoronto.ca.

Professional

As ***Professionals*** primary health care pharmacists honour their roles as self-regulated professionals through both individual patient care and fulfillment of their professional obligations to the community and society at large.

Description:
PHCPs conduct themselves professionally in all situations related to patient care and interprofessional collaboration. Pharmacists are accountable to patients, health care professionals and the primary health care system. PHCPs support the evolving role of the pharmacist within the primary health care system.

As **Professionals** primary health care pharmacists:[1,6]

6.1 Demonstrate professionalism throughout patient encounters.
- 6.1.1 show respect for patients by acknowledging the patient as a person;
- 6.1.2 integrate patient's preferences related to culture, beliefs and practices;
- 6.1.3 involve the patient in decision-making, respecting their right to make their own choices; and
- 6.1.4 accept responsibility for recognizing and meeting patients' medication therapy needs;

6.2 Practice in an ethical manner which assures primary accountability to the patient.
- 6.2.1 maintain patient confidentiality;
- 6.2.2 fulfill requirements for obtaining patient consent;
- 6.2.3 maintain appropriate boundaries with patients;
- 6.2.4 prioritize patient needs, accept inconvenience and subordinate their personal interests to those of their patients;
- 6.2.5 ensure the continuity of patient's care, abiding by the principle of nonabandonment, and
- 6.2.6 recognize and manage ethical dilemmas including conflicts of interest.

6.3 Practice in a manner demonstrating professional accountability.
- 6.3.1 be accessible to patients and other health care professionals;
- 6.3.2 fulfill their professional tasks and commitments in a diligent, timely, reliable respectful manner;
- 6.3.3 accept responsibility for their decisions and recommendations;
- 6.3.4 employ flexible and adaptable approaches to meet the needs of patients and other health care professionals;
- 6.3.5 use health care resources appropriately, including human and financial resources;
- 6.3.6 maintain their professional composure even in difficult situations;
- 6.3.7 maintain appropriate professional boundaries;
- 6.3.8 practice within their competence and scope of practice;
- 6.3.9 comply with the legal and regulatory requirements of practice; and
- 6.3.10 keep clear, accurate and legible records that are consistent with applicable legislation, regulations, policies, standards and best practice.

6.4. *Ensure their personal competence to fulfil the evolving primary health care pharmacist's role.*
- 6.4.1 identify their limits of competence;
- 6.4.2 plan and undertake learning activities to develop and maintain competence; and
- 6.4.3 assess the impact of learning on competence and practice performance.

6.5 *Support the profession and its evolving role in the primary health care system.*
- 6.5.1. participate in education of future pharmacists by making practice-based learning opportunities available as a mentor/preceptor.

Contact natalie.kennie@utoronto.ca.

Scholar

As ***Scholars*** primary health care pharmacists have and can apply the core information required to be a medication therapy expert, and are able to master, generate, interpret and disseminate pharmaceutical knowledge.

Description:
PHCPs have a broad and deep knowledge related to conditions frequently seen in primary health care and are able to build knowledge for uncommon conditions in this environment.

PHPCs use their knowledge and skills:

- for clinical reasoning and decision-making required during daily pharmacist practice;
- to provide drug information and recommendations for common and ill-defined questions, and in areas where good evidence exists and in those of clinical uncertainty regarding medications and appropriate medication use for uptake and implementation into practice.
- to formally educate diverse audiences such as patients/families, health care professionals and students[10] regarding medications and appropriate medication use, health promotion or self-management; individually and in groups; and
- to initiate or collaborate on projects related to medication use or practice problems identified during daily pharmacy practice and the primary health care system.

As **Scholars** primary health care pharmacists:[1,7,13-18]

7.1 Demonstrate a thorough understanding of the fundamental knowledge required of pharmacists by applying this knowledge in daily practice.

7.1.1 rationalize their recommendations and decisions with appropriate, accurate explanations and best evidence;
7.1.2 rely on professional experience to develop solutions to routine, previously encountered problems;
7.1.3 utilize a comprehensive systematic process to manage complex patients or when encountering non routine situations within their practice, and
7.1.4 undertake and apply learning required to manage new problems.

7.2 Provide drug information and recommendations regarding medications and appropriate medication use for uptake and implementation into practice.

7.2.1 identify needs for information and recommendations on medications;
7.2.2 conduct a systematic search for evidence using a variety of search methods and tools;
7.2.3 critically analyze information;
7.2.4 determine plausible solutions and select the most appropriate recommendation;
7.2.5 communicate information effectively providing recommendations and rationale;
7.2.6 select, tailor and implement specific interventions to transfer information (e.g. written summaries, practice tools);
7.2.7 evaluate the usefulness of the information provided; and
7.2.8 document the information provided;
.

7.3 Formally educate diverse audiences regarding medications and appropriate medication use, health promotion or self-management.

7.3.1 identify learning needs of the audience;
7.3.2 formulate appropriate and measurable learning objectives;
7.3.3 select educational methods and media appropriate for the learners;
7.3.4 select and organize content and design educational/learning plan;
7.3.5 implement and effectively deliver their educational/learning plans; and
7.3.6 assess the outcomes of their education.

[10] Students may include pharmacy students, and interprofessional learners such as nursing and medical residents, etc.

Contact natalie.kennie@utoronto.ca.

7.4 Participate[11] in practice research.

7.4.1 identify problems related to medication use or practice and determine their relevance;
7.4.2 formulate research questions/hypotheses;
7.4.3 design projects to address research questions;
7.4.4. take part in research projects and the dissemination of results; and
7.4.5. adhere to ethical research principles.

[11] Primary health care pharmacists are expected to participate and assist in practice research, rather than independently lead these initiatives as this would require attainment of additional competencies for leadership or mastery.

 Contact natalie.kennie@utoronto.ca.

Acknowledgements

Development and Research Team
Natalie Kennie. BSc(Pharm), PharmD
Nancy Winslade, B.Sc.Phm, Pharm.D, MHPE, Assistant Professor, Faculty of Medicine, McGill University.
Barbara Farrell, BScPhm, PharmD, FCSHP
Lisa Dolovich BScPhm PharmD MSc
Derek Jorgenson, BSP, PharmD, FCSHP
Nancy Waite BScPhm PharmD FCCP
Natalie Ward BA (Hons), MA, PhD (Candidate)
Ashley Gubbels, BScPharm, BSc
Tewodros Eguale, M.D., M.Sc, PhD (c.)
Sharon Johnston, MD

Review and Rating
Feedback and ratings were received from a number of pharmacists across Canada as part of the development and validation of the primary health care pharmacist competencies. The Research and Development Team acknowledge the invaluable insight provided by these pharmacists.

 Contact natalie.kennie@utoronto.ca.

References

1. Association of Faculties of Pharmacy of Canada Educational Outcomes Task Force. The Educational Outcomes for First Professional Degree Programs in Pharmacy (Entry-to-Practice Pharmacy Programs) in Canada (Draft February 2010).

2. Royal College of Physicians and Surgeons of Canada. The CanMEDS 2005 Physician Competency Framework. http://meds.queensu.ca/medicine/obgyn/pdf/CanMEDS2005.booklet.pdf

3. Pharmaceutical Society of Australia. Competency Standards for Pharmacists in Australia 2003. http://www.psa.org.au/site.php?id=643

4. Association of Teachers of Preventative Medicine. Core Competencies in Disease Prevention and Health promotion for Undergraduate Medical Education. Academic Medicine 2000;75(7). http://www.atpm.org/resources/pdfs/CoreCompetencies.pdf

5. Cipolle RJ, Strand LM, Morley PC. (2004). Pharmaceutical Care Practice: The Clinician's Guide. McGraw-Hill: New York.

6. National Association of Pharmacy and Regulatory Authorities**,** Model Standards of Practice for Canadian Pharmacists (March 2009)
http://napra.ca/pages/Practice_Resources/Frameworks_Standards.aspx

7. National Association of Pharmacy and Regulatory Authorities, Professional Competencies for Canadian Pharmacists at Entry to Practice, second revision (March 2007).
http://napra.org/Content_Files/Files/competencies.pdf

8. Association of Faculties of Pharmacy of Canada, Educational Outcomes for a Baccalaureate Pharmacy Graduate in Canada (March 1999)
http://afpc.info/downloads/1/Outcomes_Undergrad_1998.pdf

9. Canadian Pharmacists Association. Summary: Pharmacists Prescribing Status Across Canada October 2009
http://www.pharmacists.ca/content/about_cpha/who_we_are/policy_position/pdf/PharmacistsPrescribingInCanada.pdf

10. Canadian Interprofessional Health Collaborative. A National Interprofessional Competency Framework. February 2010
http://www.cihc.ca/files/CIHC_IPCompetencies_Feb1210.pdf

11. Canadian Patient Safety Institute. The Safety Competencies – Enhancing Patient Safety Across Health Professions. August 2009
http://www.patientsafetyinstitute.ca/English/education/safetyCompetencies/Pages/KeyMessages.aspx

12. CHSP-CPhA Primary Care Pharmacy Specialty Network ListServ Archives analysis.

13. Quality Improvement and Innovation Partnership
http://www.qiip.ca/index.php

14. Health Education Network of Delaware. Responsibilities and Competencies for Health Educators. http://www.henod.org/competencies.html (Accessed February 20th, 2010).

15. National Commission for Health Education Credentialing. Responsibilities and Competencies for Health Educators. http://www.nchec.org/credentialing/responsibilities/ (Accessed February 20th, 2010).

16. Straus SE, Tetroe J, Graham I. Defining knowledge translation. CAMJ 2009;181(3-4):165-168.

17. Task Force on a Blueprint for Pharmacy. Blueprint for pharmacy: the vision for pharmacy. Ottawa (ON): Canadian Pharmacists Association; 2008.
http://www.pharmacists.ca/content/about_cpha/whats_happening/cpha_in_action/blueprint.cfm

18. Canadian Coalition for Global Health Research. Module 2. Competency in Mentoring. June 2007.
http://www.ccghr.ca/docs/Mentoring_Modules/Mentoring_Module2_e.pdf
