# Supplementary material for: Pharmacist provision of primary health care: a modified Delphi validation of pharmacists' competencies
Source: BMC Fam Pract. 2012 Mar 28;13:27. doi: 10.1186/1471-2296-13-27 (PMC3372430; doi:10.1186/1471-2296-13-27)
Supplement: Additional file 2 — Importance Rankings (rank adjusted) for Competency Sub-elements. Importance Rankings (rank adjusted) for Competency Sub-elements. The document provides importance ratings for all primary health care competency elements and sub-elements. [file 1471-2296-13-27-S2.PDF]

Appendix 2: Importance Rankings (rank adjusted) for Competency Sub-Elements

Legend: Adjusted percentage weights and ranks assigned to competency elements using I = CF where C and F contributed equally to Importance (weight)

Question	Domain	Competency Element	Competency Sub-Element	F	C	I	Rank
1	Advocate	*Promote the health of individual patients*	Facilitate patient's interaction with the health care system through advice, education and/or guidance	5.13	3.83	0.78%	54
2			Support patient's access to required health services by representing or speaking on behalf of patients	4.13	3.77	0.60%	87
3		*Promote the health of patients and patient groups within their communities*	Determine healthcare needs of local patients and patient groups and implement programs/services to address or support those needs	2.20	3.13	0.20%	133
4			Participate in health promotion activities, public health campaigns and patient safety initiatives that are directed at disease prevention, risk factor reduction and/or harm minimization	2.60	3.23	0.26%	125
5			Undertake relevant public health screening processes for early disease detection	2.47	3.03	0.21%	131
6			Assist in planning and implementing public health promotion education and awareness raising campaigns with other health professionals	1.40	2.57	0.08%	151
7		*Support the role of pharmacists in the primary health care system*	Promote the impact of the pharmacist on patient outcomes	3.33	3.34	0.36%	114
8			Promote the role of pharmacists in the development and implementation of health procedures, policies and services	2.50	3.14	0.23%	130
9	Care Provider	**A. Assess patients** *Develop and maintain professional, collaborative relationships required for patient care*	Establish and maintain a professional, caring practice environment	5.96	4.13	1.08%	21
10			Demonstrate that the patient's goals are the priority	5.67	4.08	1.00%	27
11			Determine when it is ethically and professionally appropriate to involve caregivers	4.71	4.04	0.81%	49
12			Acknowledge and respect the roles and responsibilities of the	5.75	3.88	0.89%	34

			pharmacist, the patient and/or caregivers, and the patient's other health care professionals				
13		*Elicit and complete an assessment of required information to determine the patient's medication-related and relevant health needs*	Elicit the reason(s) for the patient's visit or referral to the pharmacist	5.58	3.71	078%	52
14			Obtain and evaluate relevant history from the patient, his/her chart, caregivers and other health care professionals (e.g. medication experience, medication history, current medication record, past and current medical history, allergies, immunizations, social drug use, previous adverse reactions, etc)	5.63	5.00	1.62%	1
15			Order and/or retrieve and assess relevant lab tests and diagnostic tests	5.04	4.54	1.15%	17
16			Perform and interpret findings of relevant physical assessment	4.75	4.29	0.95%	31
17			Complete an assessment of the patient's ability to take / use / administer his/her medications	5.21	4.75	1.33%	5
18		*Assess if a patient's medication-related needs are being met*	Evaluate the appropriateness, safety and effectiveness of a patient's medications with consideration of the patient's characteristics, values/preferences, conditions, functional capabilities, other medications and access to health care / monitoring	5.57	5.00	1.61%	2
19			Determine whether a patient is appropriately managing his/her therapy, including appropriate administration and adherence in particular for chronic disease management	5.39	4.74	1.37%	4
20			Determine whether a patient's medications are achieving the desired goals including consideration of efficacy and adverse effects	5.43	4.61	1.29%	8
21			Determine whether a patient requires medication assessment and reconciliation	5.04	4.09	0.89%	35
22			Identify a patient's medication-related needs as specific medication-therapy problems	5.26	4.04	0.91%	32
23			Determine if a patient requires additional care or services consistent with established collaborative practice agreements	4.48	3.87	0.69%	70

24		*Determine if a patient has health needs that require management*	Recognize signs, symptoms and risk factors that relate to medical or health problems that fall into the scope of practice of other health care professionals	4.83	4.39	1.02%	25
25			Recognize signs and symptoms associated with medical emergencies	3.22	5.78	1.32%	6
26			Recognize problems with activities of daily living important to the patient's well- being	4.13	4.00	0.69%	71
27		**B. Plan Care** *Refer patients for management of priority health and wellness needs that fall beyond the scope of practice of pharmacists*	Refer patients for management of priority health and wellness needs that fall beyond the scope of practice of pharmacists	3.55	3.91	0.56%	95
28		*Develop a shared plan of care that addresses a patient's medication-therapy problems and priority health needs*	Prioritize a patient's medication-related needs	5.50	4.05	0.95%	30
29			Establish goals of drug therapy with the patient (desired endpoints, target values and timeframes for medication therapies)	5.27	3.91	0.84%	46
30			Assess alternative strategies and negotiate the therapeutic option best suited to the patient in collaboration with the patient and other health care professionals	5.23	3.68	0.72%	66
31			Integrate the recommended therapeutic options for a patient's medication-related needs into a co-ordinated plan	5.09	3.86	0.79%	51
32			Determine monitoring parameters for desired therapeutic endpoints and potential adverse effect, specifying target values and start, frequency and end time-points for monitoring	5.18	3.95	0.85%	42
33			Decide specific actions to be taken by the pharmacist as necessary for the management of medication-related needs, specifically determining whether it is appropriate to: • dispense a medication according to a new prescription; • dispense an authorized refill of a medication; • authorize an extension of refills of a medication; • modify a patient's medications;	5.00	4.09	0.89%	37

			• recommend changes in medications;<br>• prescribe medications or therapies;<br>• administer a medication, and/or;<br>• refer to other health care professionals for assessment and management				
34			Determine if a patient requires information to facilitate his/her management of needs related to activities of daily living, health promotion or well-being	4.82	3.32	0.51%	101
35			Negotiate the care plan responsibilities of the pharmacist and patient, and when other health care professionals should be contacted	4.82	3.45	0.57%	93
36		*Implement the care plan*	Undertake specific actions as specified in the care plan (e.g. prescribing, ordering labs)	5.23	4.23	1.00%	26
37			Educate a patient regarding the care plan to facilitate understanding and adherence	5.23	4.00	0.88%	38
38			Facilitate the continuity of care through referral and communication with relevant care providers	4.86	3.77	0.71%	67
39			Convey information on maintaining and promoting health and self-management	4.73	3.36	0.52%	98
40			Convey information about available social support services to assist with daily living	3.41	3.18	0.33%	118
41			Schedule required follow-up in accordance with a patient care plan	5.27	3.55	0.66%	77
42		**C. Follow-up and Evaluate**<br>*Elicit clinical and / or lab evidence of patient outcomes*	Evaluate the efficacy of the care plan relative to the desired goals	4.91	3.82	0.74%	64
43			Evaluate the safety of the care plan including the presence of adverse drug reactions or effects	5.14	4.50	1.15%	18
44			Recognize, disclose and manage adverse drug events	4.50	4.82	0.88%	11
45			Identify any medication errors or close calls	3.77	5.05	1.11%	19
46			Determine changes in pharmacotherapy that are required	5.23	4.27	1.03%	24
47		*Assess and manage patients' new*	Assess and manage patients' new medication-related needs	4.70	4.10	0.84%	45

		medication-related needs.					
48		**D. Document** *Document their patient care activities*	Document in a timely, retrievable, usable manner	5.70	4.35	1.17%	13
49			Maintain, clear, accurate and appropriate records for all patient encounters	5.65	4.17	1.05%	23
50			Document their decisions/actions, supporting patient and related information and their interpretation of this information	5.52	4.22	1.05%	22
51			Document communication with other health care providers and health/social agencies	4.96	4.09	0.88%	39
52			Document the reporting of adverse events	3.57	4.26	0.70%	68
53	**Collaborator**	*Function as members of teams*	Clarify roles, responsibilities and expertise of other professions, identifying overlaps and gaps	2.63	2.89	0.20%	134
54			Recognize and respect the roles, responsibilities and competence of other professions	4.84	3.21	0.48%	107
55			Accept leadership roles where appropriate	3.21	2.89	0.25%	128
56			Actively make their expertise available to others and willingly agree to share relevant information, using language that can be understood by all	5.05	3.37	0.56%	96
57			Make their points of view known, listen to and respect the opinions of others, defend points of view if necessary	4.47	3.42	0.51%	100
58			Contribute to planning, organizing and performing the patient care to be provided and evaluating the results	3.95	3.47	0.47%	109
59			Respect the rules established by the group	5.79	3.21	0.57%	92
60			Help maintain a healthy work environment and assist with conflict management	5.32	3.32	0.57%	94
61			Support continued efforts of the group by providing positive feedback, including evidence of progress and impact	4.11	2.89	0.31%	120
62			Facilitate discussion and interaction among team members	4.58	2.89	0.35%	117
63			Participate and be respectful of all members' participation in collaborative decision-making	5.37	3.16	0.51%	103
64			Adapt their roles in teams and networks of care to the circumstances and requirements	5.05	3.16	0.48%	105

65		*Work collaboratively with the patient and his/her health care professionals to provide care and services that facilitate management of the patient's health needs*	Develop and maintain effective collaborative working relationships with a network of local health care professionals including the family physicians and those within the pharmacy profession	5.05	3.53	0.62%	83
66			Ensure that the care and services that the pharmacist accepts to provide patients is consistent with laws / regulations relevant to collaborative care	5.47	4.42	1.17%	12
67			Ensure their attainment and maintenance of training / certification / credentials required to provide collaborative care or to fulfill medical directives / delegation	3.42	4.32	0.69%	72
68			Ensure legality of collaborative practice agreements / medical directives / delegation agreements	2.37	4.37	0.49%	104
69			Plan the provision of care in a coordinated fashion	3.89	3.63	0.52%	99
70			Provide agreed upon care and services	5.42	4.16	1.00%	28
71			Communicate with other health care providers in a collaborative, responsive and responsible manner	5.67	3.79	0.83%	47
72			Seek out, integrate and value, as a partner, the input and the engagement of the patient//family/ community and health care team in designing and implementing care/ services	4.11	3.74	0.58%	90
73			Identify and act on sub-optimal care issues, safety issues, priorities and adverse events in the context of team practice	3.47	4.16	0.64%	80
74	**Communicator**	*Communicate non-verbally and verbally with others*	Demonstrate active listening skills and respond appropriately	5.89	3.95	0.96%	29
75			Exhibit empathy, tact and respect in their dealings with others	5.89	3.74	0.84%	43
76			Recognize and respect cultural diversity and health literacy needs	5.11	3.74	0.73%	65
77			When speaking, use organized processes and appropriate, precise expressions and vocabulary	5.79	3.63	0.77%	56
78			Tailor the content of their communication to specific contexts and audiences	5.58	3.63	0.74%	63
79			Adapt their communication techniques to facilitate efficient and effective clinical encounters	5.63	3.47	0.67%	74

80			Convey information in a way that is understandable, and that encourages discussion and participation in decision-making	5.74	3.63	0.76%	58
81		*Communicate in writing*	Write clearly, and efficiently using organized processes and appropriate vocabulary	5.53	3.68	0.76%	59
82			Correctly apply the rules of syntax, grammar and punctuation	5.32	2.63	0.32%	119
83			Tailor the content of their arguments to specific contexts and target audiences	5.00	3.16	0.47%	108
84		*Present information*	Appear comfortable, engage the audience, use appropriate tone and pace, and use nonverbal language appropriately	2.47	2.79	0.17%	136
85			Organize presentation and sets and adheres to appropriate time limits	2.00	2.53	0.11%	146
86			Respond to and manage interaction with the audience	2.00	2.74	0.13%	142
87		*Use communication technology*	Use effective communication skills regardless of the media employed including effective use of visual and educational aids	3.58	2.74	0.24%	129
88			Effectively use information and communication technology to improve interprofessional patient-centred care	3.79	3.21	0.37%	112
89		*Communicate effectively in special high-risk situations and address challenging communication issues.*	Recognize and effectively manage patient barriers to communication and understanding (e.g. cognition, mental illness, cultural, language, socioeconomic status, hearing and sight impairment, etc)	4.68	3.95	0.76%	57
90			Engage patients or substitute decision-makers in a discussion of risks and benefits of treatments and to obtain informed consent	4.37	4.05	0.76%	61
91			Communicate to others the urgency of a clinical situation	4.05	5.00	1.17%	14
92			Employ appropriate communication approaches in high-risk situations, such as in clinical crises, emotional or distressing situations, and conflict	2.89	4.89	0.79%	50
93			Use appropriate communication approaches to provide safe transfers, transitions of care and consultations among health care providers, within and between sites of care	3.42	4.05	0.59%	88
94	**Manager**	*Manage their personal practice*	Manage their time to balance patient care, workflow and practice requirements	5.32	3.53	0.66%	79

95			Adapt their practice to fulfill evolving professional roles	4.16	3.42	0.48%	106
96		*Support the sustainability of their practice*	Appropriately record workload and, where non-salaried, bill for patient care and professional services	4.05	2.68	0.26%	126
97			Adapt their practice, providing new or emerging services as consistent with patient need and/or management's practice change plans	1.74	3.21	0.17%	137
98			Incorporate the use of technologies to improve efficiency and effectiveness of their practice	2.79	3.11	0.25%	127
99			Ensure patient and team awareness of documented impact/value of their care and professional services	2.21	2.95	0.18%	135
100		*Participate in the development of policies and procedures supportive of the safe and effective use of medications and the provision of quality primary health care*	Develop and practice according to policies and procedures that support improved access to health services (e.g. collaborative practice agreements, medical directives)	3.00	3.63	0.40%	110
101			Develop and adhere to delegation and workflow policies that ensure efficient use of human resources	3.68	2.95	0.29%	121
102			Develop and incorporate processes supportive of patient self-management into their practice	3.32	3.05	0.41%	123
103		*Recognize the occurrence of errors and unsafe practices and respond effectively to mitigate harm to the patient, ensure disclosure, and prevent recurrence*	Recognize, disclose, manage and report errors, incidents and unsafe practices	2.47	4.74	0.63%	82
104			Participate in timely event analysis, reflective practice, and planning for the prevention of recurrence	1.74	4.37	0.36%	115
105		*Participate in quality assurance and improvement programs*	Ensure the care they provide is timely, cost effective and results in positive impact on care processes and/or patient outcome	2.84	3.26	0.29%	122
106			Participate in planning for implementation and evaluation of services/programs to meet patient's needs and/or improve the quality of care provided, including strategies to identify and overcome barriers, and to capitalize on facilitators	1.56	3.22	0.15%	138
107	**Professional**	*Demonstrate professionalism throughout patient encounters*	Show respect for patients by acknowledging the patient as a person	5.94	3.61	0.78%	53
108			Integrate patient's preferences related to culture, beliefs and	5.72	3.50	0.69%	69

			practices				
109			Involve the patient in decision-making, respecting their right to make their own choices	5.67	3.78	0.83%	48
110			Accept responsibility for recognizing and meeting patients' medication therapy needs	5.78	3.83	0.87%	40
111		*Practice in an ethical manner which assures primary accountability to the patient*	Maintain patient confidentiality	5.94	4.39	1.25%	9
112			Fulfill requirements for obtaining patient consent	4.94	3.89	0.77%	55
113			Maintain appropriate boundaries with patients	5.78	4.22	1.11%	20
114			Prioritize patient needs, accept inconvenience and subordinate their personal interests to those of their patients	5.22	3.56	0.66%	78
115			Ensure the continuity of patient's care, abiding by the principle of nonabandonment	5.22	3.94	0.85%	41
116			Recognize and manage ethical dilemmas including conflicts of interest	3.94	3.89	0.62%	85
117		*Practice in a manner demonstrating professional accountability*	Be accessible to patients and other health care professionals	5.44	3.39	0.61%	86
118			Fulfill their professional tasks and commitments in a diligent, timely, reliable respectful manner	5.89	3.83	0.89%	36
119			Accept responsibility for their decisions and recommendations	5.94	4.33	1.21%	10
120			Employ flexible and adaptable approaches to meet the needs of patients and other health care professionals	5.67	3.39	0.64%	81
121			Use health care resources appropriately, including human and financial resources	5.33	3.44	0.62%	84
122			Maintain their professional composure even in difficult situations	5.17	3.94	0.84%	44
123			Maintain appropriate professional boundaries	5.78	3.61	0.76%	62
124			Practice within their competence and scope of practice	5.94	4.72	1.49%	3
125			Comply with the legal and regulatory requirements of practice	5.83	4.50	1.30%	7
126			Keep clear, accurate and legible records that are consistent with applicable legislation, regulations, policies, standards and best practice	5.72	4.33	1.17%	15

127		*Ensure their personal competence to fulfil the evolving primary health care pharmacist's role*	Identify their limits of competence	5.06	4.56	1.16%	16
128			Plan and undertake learning activities to develop and maintain competence	2.44	3.39	0.27%	124
129			Assess the impact of learning on competence and practice performance	1.94	2.83	0.14%	140
130		*Support the profession and its evolving role in the primary health care system*	Participate in education of future pharmacists by making practice-based learning opportunities available as a mentor/preceptor	1.89	2.83	0.14%	141
131	**Scholar**	*Demonstrate a thorough understanding of the fundamental knowledge required of pharmacists by applying this knowledge in daily practice*	Rationalize their recommendations and decisions with appropriate, accurate explanations and best evidence	5.22	4.06	0.91%	33
132			Rely on professional experience to develop solutions to routine, previously encountered problems	4.83	3.72	0.68%	73
133			Utilize a comprehensive systematic process to manage complex patients or when encountering non-routine situations within their practice	4.22	4.11	0.76%	60
134			Undertake and apply learning required to manage new problems	3.67	3.78	0.54%	97
135		*Provide drug information and recommendations regarding medications and appropriate medication use for uptake and implementation into practice*	Identify needs for information and recommendations on medications	4.72	3.72	0.67%	75
136			Conduct a systematic search for evidence using a variety of search methods and tools	3.61	3.72	0.51%	102
137			Critically analyze information	3.78	3.83	0.57%	91
138			Determine plausible solutions and select the most appropriate recommendation	4.39	3.83	0.66%	76
139			Communicate information effectively providing recommendations and rationale	4.50	3.61	0.59%	89
140			Select, tailor and implement specific interventions to transfer information (e.g. written summaries, practice tools)	3.61	3.22	0.36%	116
141			Evaluate the usefulness of the information provided	3.89	3.22	0.39%	111
142			Document the information provided	4.44	3.00	0.37%	113
143		*Formally educate diverse audiences*	Identify learning needs of the audience	1.61	2.83	0.12%	145

144		*regarding medications and appropriate medication use, health promotion or self-management*	Formulate appropriate and measurable learning objectives	1.50	2.61	0.09%	149
145			Select educational methods and media appropriate for the learners	1.56	2.89	0.12%	143
146			Select and organize content and design educational/learning plan	1.50	2.72	0.10%	147
147			Implement and effectively deliver their educational/learning plans	1.56	2.89	0.12%	144
148			Assess the outcomes of their education	1.50	2.72	0.10%	148
149		*Participate in practice research*	Identify problems related to medication use or practice and determine their relevance	2.28	3.11	0.21%	132
150			Formulate research questions/hypotheses	1.44	2.50	0.08%	152
151			Design projects to address research questions	1.11	2.61	0.07%	153
152			Take part in research projects and the dissemination of results	1.28	2.67	0.08%	150
153			Adhere to ethical research principles	1.61	3.17	0.15%	139
